# Installed Base as a Facilitator for User-Driven Innovation: How Can User Innovation Challenge Existing Institutional Barriers?

**DOI:** 10.1155/2012/673731

**Published:** 2012-12-06

**Authors:** Synnøve Thomassen Andersen, Arild Jansen

**Affiliations:** ^1^Department of Business and Tourism, Finnmark University College, N-9509 Alta, Norway; ^2^Section for e-Government Studies, Department of Private Law, University of Oslo, 0130 Oslo, Norway

## Abstract

The paper addresses an ICT-based, user-driven innovation process in the health sector in rural areas in Norway. The empirical base is the introduction of a new model for psychiatric health provision. This model is supported by a technical solution based on mobile phones that is aimed to help the communication between professional health personnel and patients. This innovation was made possible through the use of standard mobile technology rather than more sophisticated systems. The users were heavily involved in the development work. Our analysis shows that by thinking simple and small-scale solutions, including to take the user's needs and premises as a point of departure rather than focusing on advanced technology, the implementation process was made possible. We show that by combining theory on information infrastructures, user-oriented system development, and innovation in a three-layered analytical framework, we can explain the interrelationship between technical, organizational, and health professional factors that made this innovation a success.

## 1. Introduction

Most innovations take their point of departure from a technological perspective, not least when it comes to the health sector. The main message is that ICT can solve the great challenges we are facing in transforming the health sector and make it more efficient and citizen oriented [1]. However, the implications of this perspective very often seem to entail expert-driven, top-down development work, where neither citizens nor health professionals are involved. However, improving health care is not primarily a matter of technology. Close collaboration with health care providers and cooperation between health professionals and patients are essential factors in achieving better health care. The mobilization of patients' own resources as well as family and community resources can contribute significantly to the healing process [2, 3].

Our case is an example of a user-driven, bottom-up development process, in which local professional along with organizational needs and user interests have strongly influenced the development process. The catalyst for this process was the introduction of a new health program based on the Parent Management Training-Oregon (PMT-O) model. This is a treatment and prevention program for families with children displaying antisocial behaviour (PMT-O is based on “Social interaction learning theory”, developed by Patterson and co-workers at Oregon Social Learning Center. PMT-O is a detailed program designed to improve parenting practices and indirectly reduces antisocial behavior in the children). An important part of this project has been the development and implementation of an appropriate technical solution based on mobile phones used to help care providers and patients in their communication and information handling routines. The users were heavily involved in the design work as they were familiar with the technical platform to be used. Accordingly, the innovation has primarily been an organizational transformation, strongly supported by technical development work. A challenge, however, was to obtain acceptance for this type of user-driven system development and implementation in a strongly institutionalized health sector. 

Our research focus is how the innovation process has taken place and the factors that have been crucial. We claim that the cardinal moment in the design process was the decision to break with the existing technical and organizational power structure and instead to rely on local resources along with the mobile phone infrastructure and services. This implied a move to a new technological platform, the development of a new application, the establishment of a technical support group, and the building of a new health care organization.

 The aim of this paper is to contribute to the understanding of how user-driven innovations can be stimulated and in particular how the installed base of the infrastructure may act as a facilitator for user-driven innovation. Our research question is *How to facilitate user-driven innovations in an institutionalized environment*.

### 1.1. Structure of the Paper

This paper is structured as follows: first, a presentation of the theoretical basis in Section 2, thereafter a presentation of the method, while Section 4 presents the case and empirical basis. The analysis and discussion of the findings are presented in Sections 5 and 6, followed by our conclusions.

## 2. Theory

The theoretical basis for this paper is (i) theory on information infrastructure, (ii) principles of user-centred system development, and (iii) innovation theory. Our theoretical contribution is derived by bridging knowledge from different academic fields, which can improve our understanding of how users can contribute in the development and diffusion of new technology in health care.

### 2.1. Information Infrastructure

Information Infrastructures (II) are conceived as having complex, unbounded, and sociotechnical characteristics [4–6]. Hanseth and Lyytinen, [7] define an II as “a *shared, evolving, heterogeneous installed base of IT capabilities among a set of user communities based on open and/or standardized interfaces.”* Information infrastructures, when appropriated by a community of users, offer *shared *resources for delivering and using information services to its users. We now see how traditional information systems are being transformed into IIs by their advances in reach, range, and integration into complex corporate wide and industry wide information infrastructures [8]. We regard these information infrastructures as a new class of IT systems which also need to conform to a set of design requirements that are different from those of traditional information systems [9].

The installed base is an essential part of an information infrastructure, which is always built on or extended from its existing base. An II combines and draws upon heterogeneous and diverse components that are not under the control of one designer [4, 10–12]. The installed base can be understood as a heterogeneous “network” of technical, organizational, legal, financial, and human components and also as the accrued continuous practices and technologies that are institutionalized in the organization [13]. Therefore, the entire infrastructure cannot be immediately changed; however, new components can be integrated with the old. Hanseth and Lyytinen [7] claim that “Overall, the evolution of infrastructures is both enabled and constrained by the installed base, that is the existing configuration of II components.” Whatever is added needs to be integrated and made compatible with the existing base. This sets up demands for horizontal and/or backwards compatibility and imposes constraints on what can be designed at any time. Accordingly, “II evolution is path dependent and shaped by neighbouring infrastructures, existing IT capabilities, user and designer learning, cognitive inertia, and so forth.” (ibid).

### 2.2. User-Driven System Development

Our second theoretical leg comprises theories on user-driven system development or participatory design which have been much influenced by the Scandinavian tradition in information system research, grounded in sociotechnical thinking and action research from the 1960s and 70s [14, 15]. Participatory design is an approach to design attempting to actively involve all stakeholders (e.g., employees, citizens, end users) in the design process in order to ensure that the end product meets their needs and is usable, as described in the ISO standard *human-centred design for interactive systems* (ISO 9241-210, 2010). Since the 1900s, user-driven development included iterative processes and agile development processes based on early planning and short iterative cycles with possibilities for interaction with the users during the whole development process [16–19]. Another tradition is human-computer interaction, which involves the study, planning, and design of the interaction between users and computers and focuses on user interface and usability [20, 21]. Involving the users in system development work in general may have different aims, such as allowing the users to influence the technical design, to focus on changes in work tasks and practices, to teach and motivate the user, or to achieve more overall political goals such as organizational restructuring.

Many methods and techniques may be applied, such as the use of prototypes, either as a model or as a concept in the design process, or as a first version of the final product or process [22]. The Rational Unified Process (RUP) emphasizes iterative, incremental processes [23, 24]. RUP applies *use case* as technique, defining the interactions between a role/actor (human, external system) and a system, to reach a goal [25].

### 2.3. Innovation

Our third perspective is that of innovation. Rogers [26] points out that an innovation may be broadly defined as a process, knowledge, or technology that brings about something new. This may lead to a more active role for the users in the innovation process. Like other types of innovations, IT innovations are developed on the basis of different sources that cover a wide range of activities in the IT value chain [27]. This implies that an IT innovation must negotiate a complex ecology of multiple types of innovative events [27, 28]. Choice of technology is an important part of many projects, but strategies, processes, people, and culture are also important aspects. Users are often familiar with social media like Facebook and Twitter, and they expect to find similar utilities in other settings, not least at the workplace. It is important to develop innovation strategies that address user's needs, possibilities, and necessary utilities. “As toolkits are more generally adopted, the organization of innovation-related tasks seen today, especially in the field of custom integrated circuit development, will spread and users will increasingly be able to get exactly the products and services they want—by designing them for themselves” ([29], page 256). This concept illustrates the terms “user-centred innovation” and “lead users” in democratization of innovation [30]. Further he predicts “that the user's ability to innovate is improving radically and rapidly as a result of the steadily improving quality of computer software and hardware, improved access to easy-to-use tools and components for innovation, and access to steadily richer innovation commons” ([30], page 21).

Innovation is also described as a network activity in which the traditional conception of organizations has been emphasized to a lesser extent. By focusing on the “combinational” and the “organic” model of innovation, in which various competences and developments interrelate, innovation becomes construable as what it is, namely, as a social process [31]. Tuomi held that two of the driving forces for innovation today are new technological possibilities based on ICT as well as the need for more individual user requirements.

### 2.4. Our Research Framework

As a point of departure, we consider the information infrastructure as heterogeneous, modular, and layered, where the user applications and surrounding organizational and legal context are important parts of its installed base. In our case, the innovation included technical aspects, usability, and organisational elements. We will have to analyse the innovation processes at three levels, as illustrated in Table 1.

The infrastructure level addresses the specific characteristics of the existing and the new installed bases, and in particular how it influenced the change process related to the technical and organisational innovations. Furthermore, we will identify the critical factors and processes such as basic function services, types of applications, and typical users.

The middle application development level addresses the application development and the user involved, with a view to understand the different phases in the system development process and how they involved various user groups having different background/experience, roles, and interests and how it was possible to solve the potential conflicts in this work. Other factors may be the different actors, their functions and roles, and, finally, conflicting interests. 

The top, organizational level includes provision of health services, and so forth. Our focus aims to understand the organizational change processes that have taken place in the provision of psychiatric health service, including changes in professional work, in relations between professionals and users, as well as institutional and professional conflicts. Critical factors are the institutional context, changes in the organization, important actors and power structures, and so forth.

 Our overall research question is “*how to facilitate user-driven innovations in a professional, institutionalized environment”,* and the analysis will focus in particular on the interactions between the various factors at these different levels. 

## 3. Method 

A qualitative research method in the interpretative tradition of IS studies [32–34] has been applied. Qualitative research is designed to aid researchers in understanding persons and the social and cultural context in which they are situated [35], which has been of particular importance in our case. The data collection has followed the progression of the project. One of the authors was both researcher and project manager since start-up in 2006 and has therefore been directly involved in the development of the innovation project throughout a large number of project activities. The author's involvement alternated between participant observation and active involvement. This entailed certain challenges in balancing the pursuit of research interests with efforts to achieve the goals of the project. Walsham [36] points out the importance of the interpretive researchers having insight into their own roles in the complex process that occurs between people. In our case, the researcher has been engaged in both the data collection and their interpretation, and these activities have inevitably involved the researcher's subjective assessments. It has therefore been important to use an open dialog to handle this dual role of being both a researcher and a project manager. In addition, a professional distance to the patients was maintained by channelling all contacts with them via the therapists and the professional teams. 

Our study builds on observations, interviews, and studies of documents throughout the entire project period. The participation in 63 formal meetings also provided a comprehensive and important source of data material. Data collection was done through anonymous questionnaires sent to the families, interviews with all members of the ambulant teams and interviews with user representatives, observations related to work in the techno group, the project group and steering group, and observations of children from one of the pilot municipalities. We have primarily interviewed health workers and the user groups that represented the children and families. All interviews were transcribed (the text is translated from Norwegian into English by one of the authors). Another important data source came from our observations as participants. The observation of the different users was done over a long period of time. In addition we have analysed a large number of minutes from meetings, reports from workshops, user training, e-mail correspondence, and reports.  Table 2 shows the various activities associated with data collection, collection/data techniques, and the total number in relation to the individual activities. All types of data are linked with one another and have been used in the analysis.

## 4. The Case

The empirical base for our study was the project called “Come Here!—ambulant teams and technology” (the Norwegian title: “Kom hIT—ambulante team og teknologi” includes a pun through capitalising IT in the word “hit” (meaning here)) which introduced a new health program in Finnmark based on the PMT-O model; an outpatient treatment model for parents with children that can be difficult to bring up. The research has been process oriented since it was based on observations of the actors, their project setting, and technical development work over time including the design of mobile application and organizational changes. Finnmark County, with its less than 73,000 inhabitants, is very sparsely populated. Approximately 20 000 persons are under the age of 20 (in 2007) and of these, 950 children under 18 were receiving daily treatment in clinics for children and youth psychiatry. Distances are great between communities, and most inhabitants also have long distances to travel to get to the nearest hospital or medical expert. The broadband and mobile infrastructure in the county is unevenly distributed; some areas are well covered by both broadband networks and telephone networks, while others are practically without any coverage at all.

The project was established 1 January 2006 following the decision of 1 July 2005 by the County Health Authority to close down the only existing psychiatric hospital. The functions served by the hospital were to be compensated by a decentralized treatment model, where ambulant teams would conduct and support home-based treatment for both families and children between the ages of 6 and 12. The goal in offering this decentralized treatment model was to help parents manage their children's behaviour. This method focuses on social skills and cooperation and is designed to prevent, reduce, and reverse the development of moderate to severe behavioural problems in children. It was also designed to strengthen the interdisciplinary cooperation between different professions and levels in the health organizations. Another goal was to increase efficiency in the treatment by applying ICT tools.

**Figure 1:**
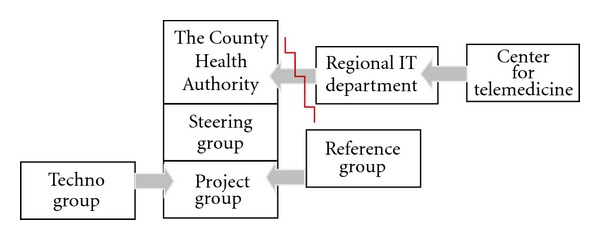
Project organization.

The project was established and included a steering group, a project group, and a reference group anchored at the top level management of the Health Finnmark Authority (see Figure 1). The first task was to analyse the roles and interests of the different users, professionals, administrative staff, and families. The project group was supposed to give the steering group advice in deciding the treatment method as well as the technical solution that needed to be developed. 

During the first period, the project group cooperated with the County IT department. However, this IT department formally withdrew from the project nearly a year after the project had started, due to the reorganization of the department effective 1 January 2006 and its integration into the Northern Norway Regional Health Authority (RHF). This was a centralization of the three county IT functions into one regional IT department linked with the Centre for Telemedicine (NCT) (Norwegian Centre for Integrated Care and Telemedicine (NCT), which is a unit under University Hospital in Tromsø) in Tromsø.However, as a consequence of this reorganization, it became difficult to cooperate with this central IT department; despite multiple enquiries, the project team never received any documentation of the existing information infrastructure (the Norwegian Health Network; in Norwegian: “Norsk helsenett”, see http://www.norsk-helsenett.no/, which is a national broadband network connecting all health institutions). Such information was essential for the progress of the project. The result was a break between the project and the IT department, and a local techno group was established. Figure 1 illustrates the project organization. The steering and project group was formally subordinate to the RHF. The reference group and the techno group were mandated by the steering group, while the regional IT department was part of the Centre for Telemedicine, responsible for technical support and services (the IT department as a contractor of ICT services to the specialist health care in Health North shall provide the clinics and others with the most appropriate ICT systems, and the department aims to become the most preferred ICT contractor based on costs, quality, cooperation, knowledge, and experience).The conflict between the County Health Authority and the regional IT department is indicated by the red mark, which resulted in a schism entailing consequences for funding as well as for the design of the system.

The formal project organization clarifies responsibilities and work processes and is characterized by work sharing, leadership, and seeing users as a resource to reach the project aim.

### 4.1. The Implementation of the Professional Treatment Model

The basic idea of the PMT-O model is close cooperation between professionals' ambulant teams and the families. During an initial meeting between the ambulant teams and the parents and their child, the goals to be reached during the treatment will be defined and prioritized. Furthermore, the teams will negotiate the specific patient behaviour that should be encouraged or discouraged through the treatment, for instance their behaviour during meals or when going to bed. The child's rewards in relation to these action points are then defined; as well as how many score points can be earned for certain types of behaviour. The purpose of the treatment is to ensure that the child and his/her parents are able to reestablish a positive relationship so that oppositional behaviour can be dispelled and a positive development can be fostered. The ambulant teams will frequently visit the parents; in between, the parents, in cooperation with the child, are supposed to frequently register the behaviour and assign a score that is during every meal or every evening when the children go to bed. A report is created from the treatment log, which constitutes the basis for the interaction between the family and the ambulant team. The specific goals in the project were as follows.

1. The mobile teams shall make sure that the child is getting help where he/she lives.
2. The mobile teams shall make sure that the child and his/her family or relatives get adequate qualitative and testable verifiable methods for treatment.
3. The mobile teams will contribute to strengthen the cooperation and interaction between children, their family/relatives, the school, and the health and social services in their home community, which consolidates (assures) overall good quality.

### 4.2. The Shift of Technical Platform

**Figure 2:**
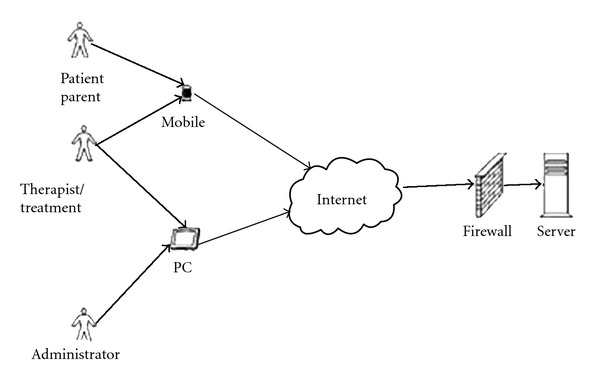
Schematic description of the new solution.

Initially, some parents would fax the completed forms to the team, while others kept these until the next time they interacted with the team. It was felt, however, that this collaboration would benefit from more frequent reporting as well as enabling easier and more frequent interaction. The aim of the project was to improve these communication patterns by introducing tools that allowed parents to continuously report the behaviour, thus enabling the ambulant team to monitor progress on an on-going basis. Accordingly, an important part of the innovation project was to choose and adapt the most appropriate technology for supporting the treatment. Two important decisions had to be made: (i) the choice of a technical platform and (ii) the overall design of the technical solution. The users, the health care workers, and the families felt that there was a need for technology that could take advantage of the existing broadband networks and telephone networks. A mobile phone platform was found adequate for supporting health workers and patients in their communication and information during the treatment. The use of mobile telephones was expected to result in closer followup of the families, in reduced travel activity and, additionally, in a cost savings [37, 38]. The solution, as illustrated in Figure 2, includes the use of both the new mobile network and the Internet. However, the Norwegian Health Network is not included in this new solution. The infrastructure is practically “invisible” to the users, except for the mobile phone network. This solution benefitted from the fact that all user groups (families, the ambulant teams and others) were already a part of this “new” installed base in being active and experienced mobile phone users. A key factor was therefore the use of the existing installed base of the mobile phone network. The ambulant teams can access the information on the server (which is located at the vendor's site) through Internet (via VPN channels) (As this is outside the firewall of the secure health-care network, there is no direct import of data into the main patient record application, but it is possible to cut and paste information from the application into the “CYP Data”, which is the main patient record application in use in the health care sector. In order to comply with national safety requirements and standards, a risk analysis of the solution was conducted by the Norwegian Centre for Informatics in Health and Social Care AS (KITH), see http://www.kith.no/. There is no local caching, so no sensitive data is saved on the phones.). The link to the Health Network has been established though a gateway between the two networks. The new solution is illustrated in Figure 2.

### 4.3. User-Driven Application Development

The choice of a different technology from what had previously been considered entailed new challenges for the project, not least in terms of the design and implementation of a new solution. The techno group that was established included representatives from ambulant teams, user organizations, and families as well as the project manager and the system supplier. The ambulant teams were supposed to have the opportunity to retrieve information registered about the children and families or to generate new forms via the PC. Another intention was to allow for messages concerning behavioural situations that could be sent and stored in a separate report file.

**Figure 3:**
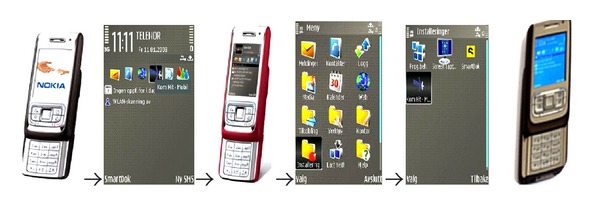
Examples of the user interfaces and interaction sequences.

The application was implemented on Nokia E65 phones that were distributed to project participants. The user interface face of the application is a screen image similar to the PMT-O paper forms used to register the results on specific action points regarding the child's problems, see Figure 3. The application is general and flexible in order to allow every child and family to adapt it to individual treatment plans. Some details of the user interface of the technical solutions are listed below:

- logging-in via mobile, entering of a user name and emailing this as an SMS text message,
- receiving an SMS text message with a password to be used in order to access the system,
- viewing the system's screen display and menu selections,
- filling out the reward form through the use of text, numbers or symbols or making changes as needed through the use of the functions Add, Update information, or Add date or ticking off to indicate one of the five steps,
- sending the form via the mobile telephone.

The users were involved in all parts of the development work, from planning to design and use. The term users in this case refers to health care workers, the project's team members, and psychiatric specialists as well as the families. Preliminary versions of the application were tested among children/adolescents and their families and the ambulant teams in various pilot municipalities during the project period.

This design of user participation must comply with laws and regulations in Norway. To support user groups' interests and rights, adequate procedures were set up for the involvement of different user groups and consultation processes. In this user-driven project, it was essential to establish several small user groups on different levels and across different professional and user interests. Large groups were considered inefficient. Some examples are from the development work. 

In order to get user involvement in the design, the supplier and techno group agreed on drawing figures as examples of how the applications would look and on using existing forms as a starting point. It was crucial that the solution would be easy to use for all user groups. The users representing the parents were of the opinion that it is challenging for families to handle psychological problems, and one participant said:

*If we have to use technology as a part of the treatment, it has to be easy to use. The solution has to have general, self-explanatory applications, and so forth, otherwise it will not be used…! *

*What about giving children, adolescents and parents the opportunity to view forms they filled out earlier? *

Another member added:

*Yes, that is very important! The children and parents should be able to read and review previous reports.*

The supplier replied:

*Feedback is important! We need to discuss the way all of you, as participants and users, wish to use this application and the various functions. *

The project manager initiated discussions and posed questions pertaining to the different treatment activities during the development work in order to clarify what the different user groups were supposed to do at different phases of the treatment. Comments included: “*What does this form imply?*” “*Why do you want to put questions like this on the screen?*” “*Why do you prefer this particular colour?*” “*Why are you doing exactly this—is it part of the method?*.” Usability tests (using both paper based and real prototypes implemented on the phones) were conducted before the final mobile application was developed, making it easier to get acceptance for the new technical solution. However, although the project resulted in daily contact with families, the mobile solution has not resulted in less travel for the ambulant teams. Another important aim of this first phase of the project was to make sure that all parties accepted this treatment model and the new organizational structure, with treatment being given in small clinics.  Table 3 illustrates how the different user groups participated in the development work.

There were not homogeneous groups of users in the project. The users were of different ages, different sexes, and belong to different professional sectors, and so forth. These unequal user groups became important factors in the user-driven system development. The families themselves had varying social background, coming from different cultural and social traditions (Norwegian, Sami, Finnish and Russian immigrants, etc.). The different user interests and preferences had to be met carefully. Even though different professions and families having different ethnical background were involved, the project managed to handle these challenges adequately. Several families and parents participated in the user training courses along with health care workers, personnel from the kindergarten/preschool, schools, and so forth, from the different pilot communities.

### 4.4. Summary of the Development Processes, Milestones, and Decisions

**Figure 4:**
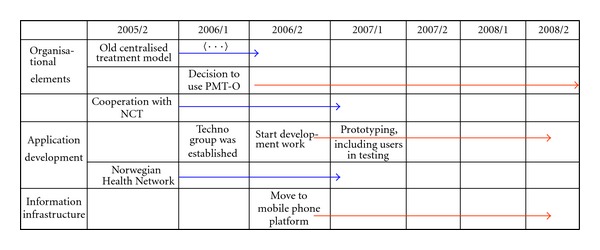
Project milestones.

The result of the project has been the development of a new technical, web-based solution along with certain organizational changes that were necessary in order to support the implementation of technology designed to be ancillary to the treatment method.  Figure 4 summarizes the milestones (critical decision points) of the project course/progress.

The figure shows the major decisions that were vital for the project.

- To use PMT-O as the treatment method, thus closing down a central institution.
- The reorganization of Health Finnmark which led to a break with the Norwegian Health Network.
- To establish the techno group in 2006, with the resulting development of the application on their own.
- User-driven development, using prototyping and testing.

The project ended on December 31 2008, and the model is to become the standard for psychiatric care for children and adolescent youth.

## 5. Critical Factors for User-Centred Innovations

Figure 4 illustrates the major milestones and decisions in the project. However, these decisions have been controversial, in the following.

1. The transformation from the existing, centralized treatment model and institutionalized practice model to a decentralized treatment model (PMT-O) implyies more user-centric practices in working routines and cooperation patterns that caused some resistance among health professionals
2. The migration from the old, centralized information infrastructure (Norwegian Health Network, NHN) to a mobile-phone-based infrastructure where the users already were part of the installed base included an alternative development approach that was user oriented and bottom-up.
3. The move from the dominating network (the Norwegian Health network) to a new mobile platform entailed development of the application using mobile phones and the mobile network along with introducing a new interaction pattern between the professional health care workers and the users.

Below, we will discuss the consequences of these decisions.

### 5.1. From Centralized to Decentralized Psychiatric Health Services

The decision by the County Health Authority to close down the only existing psychiatric hospital and to replace it with a decentralized treatment model implied a change from a traditional centralized psychiatric hospital to ambulant teams that could provide home-based treatment for both families and children, including new methods of treatment. The technical solution that was implemented was intended to support this new model and provide treatment as early as possible, while taking into account the users' cultural background, language, and so forth. This decision also entailed an organizational change: to break with existing institutional bonds (constraints) and establish a new organization.

 The County Health Enterprise decided to have strong user participation in the project, and this in turn provided an impetus for the implementation of the PMT-O. The old treatment model with its links to central clinics and hospitals, including their knowledge base, was a part of the existing information infrastructures and its installed base: work practices, professional interests and attitudes, way of thinking, and so forth. During the process of designing the technical solutions, the different user groups became motivated to accept the new treatment model, including changes in responsibilities, interaction patterns, and work routines. These experiences support Star and Ruhleder ([4], page 4) in their understanding of information infrastructure as a fundamentally relational concept that becomes infrastructure in relation to organized practice.

From the outset, this reorganization was conceived as an enabler. The link to The Norwegian Health Network (NHN) and the Norwegian Centre for Telemedicine (NCT) turned out to be a limiting factor in the development work due, for example, to the lack of information from the IT department and the fact that the NHN controlled all the technical data within their secure net. Furthermore, they suggested a technical solution based on broadband and PC and involving videoconferencing facilities; it was felt, however, that this would help users only to gain access to and become familiar with the technology. 

Traditionally, the psychiatric hospital was the only institution in the county that offered professional psychiatric treatment. This reorganization involved conflicts, since some of the health care workers hesitated to accept the change from the existing central treatment model to a decentralized model with small policlinics. This conflict was finally solved but illustrates the tensions that existed at the institutional level in terms of the changes in responsibilities and power structures. It also illustrates the sociotechnical character of technology, whereby tools and systems are closely integrated with work practice [4, 6, 39]. 

### 5.2. The Role of the Installed Base in Innovation Processes

The development project itself had significant impact on the implementation of the new organization. Strong user participation mobilized different user groups and influenced their interpretations of new mobile technologies and information systems. This user driven participation in design, development, and implementation of the new mobile solution implied that the new treatment model more easily became part of an installed base much closer to the different users' everyday life and their work patterns. We experienced that the shift from an old institutionalized structure and its installed base to the new technical platform was facilitated by the existence of the installed base linked to the mobile phone infrastructure in which the users were already enrolled. Organizational changes and the shift of technical platform became strongly interwoven in these change processes, where the different elements influenced each other, corroborating Ciborra et al. [10] who emphasized that distribution of responsibility, power, and governance in an organization is an important part of the installed base. Our case shows how the different sociotechnical components of the installed base in terms of work practices, skills, and attitudes along with the technical platform and the mobile application, and so forth were adopted and adapted through the development work, in which the involvement of the users was instrumental. User-driven innovation can thus challenge an existing installed base, or it can support the replacement of installed base. In this project, a “new” installed base became visible as a powerful factor in the implementation of the solution and contributed to a smooth transition from the existing technical and organizational base to a new infrastructure and organizational structure. Various installed bases may thus act as either enabling or constraining in system implementations. In our case, the link to the old installed base was maintained by an open gateway between the two networks, although this represented a much weaker bond than in the past. In the context of this paper, an II in the health sector, such as the Norwegian Health Network will include various networks technologies, systems, tools, and standards, but also work practices, organizational practices, and furthermore common rules and regulation that restrict or facilitate the use of the II. This illustrates that there are different actors and stakeholders who have different perspectives and have only partial control over the information infrastructure [4, 40]. 

### 5.3. From Expert to User-Driven Application Development

The initial system development approach had been based on a top-down strategy, controlled by the health administration and using NCT as experts, thus having the character of being expert driven. The existing (technical and organizational) installed base, including the telemedicine expertise (at that time (now, NCT is enthusiastic to use mobile phone technologies, see e.g., http://www.telemed.no/index.php?cat=77933)), was oriented towards using videoconference facilities, and so forth. The Norwegian Health Net offered a secure broadband network, videoconference, psychiatric information system, and so forth, representing an institutionalized solution that potentially would hinder innovation. This illustrates that we often see strong links between certain technologies and corresponding organizational structures. The county health authority used the NCT for advice to test the various kinds of video conference equipment for use in the homes; in addition, a risk analysis was conducted. However, the steering group and the project team felt that mobile technology could serve as an appropriate technical platform for this type of health service. This small scale, user friendly, and familiar mobile phone network offered a technical platform making it easier for the users to participate in the development of the application. Ciborra et al. [10] point to how different parts of an infrastructure will be under the control of specific actors. In our case, the mobile network appeared as open, allowing for development of new applications fairly easily, while the National Health Network was perceived as being difficult to access. This experience is also in line with Rolland [13, p.6], who argues that “the installed base seems to become increasingly visible as the system is embedded in an organizational context and during negotiations between different interest groups in the design phase.” Technology that should support the new activities may very well prevent such changes, and strategies for loosening what seem to be “locking” bonds are necessary to manage changes in adequate ways. The context for the innovation process was reorganization; the users were about to reorganize, to begin to become ambulant. They needed a new support tool, and they began to look at various technologies and information infrastructures with which they already were very familiar.

This development work has been similar to what we experience on the Internet today, as for example, the development of applications on smart phones, using an infrastructure with which the users already are very familiar.

### 5.4. Users Influencing Innovations in Institutional Reorganization

As stated previously, the break with the institutional links made the user-driven system of development possible. Use case and prototypes functioned as fruitful techniques, along with the information about the ambulant teams' work routines provided by their own representatives, in order to obtain first-hand information related to work practice and use of the existing system. Mumford [41] claims that user-driven system development is not a unique term. The users can be involved in several ways, and according to Mumford [41], the focus should be on what kind of user-driven participation we want and what the purpose is. The first phase focused on having the different stakeholders accept the new treatment approach and implement the organizational model. The result of this phase was that it also became easier to gain acceptance for the new technical solution. Despite the extra work involved in development, the representatives for the families expressed that they had gained more insight into the organization of the psychiatric health services offered, as well as a deeper understanding of the use of mobile telephones in practice. This user-driven system development was made possible due to the simplicity of the new technical platform and the ease of enrolling new users into the installed base. This user-oriented innovation was thus based on strong sociotechnical orientation, which involved the different user groups within all project phases, in line with Jansen [42]. There were only minor conflicts of interests, which were easily solved due to the strong involvement of users. 

Chapter 2 presented multilevel framework for understanding how the development and implementation work involved innovations at three levels: technical platform, application, and organization, which can be illustrated in Table 4. 

The analysis has shown that user involvement was made possible by the specific character of this decentralised reform process, being rooted in the local health care organisation and driven by local psychiatric specialists in close cooperation with their clients (the families) and using standard technology. Thus, we see that the success factors were as follows.

1. The acceptance of the adoption and adaptation of the decentralized treatment model (PMT-O), including the reconciling potential professional and social conflicts.
2. The establishment of a local development organization with a strong focus on user involvement.
3. The decision to use the mobile phones and the existing infrastructure, where the users were already part of the installed base, thus to build the application on a technology with which the users were already familiar.
4. A development approach based on a user-oriented, bottom-up strategy and implementation in a decentralized environment.

These experiences conform to similar efforts in technology transfer, using an appropriate technology [43] adapted to the local technical, organisational, and cultural context. While the project from the outset was strongly linked to a rather centralised organisation and technical platform, being rather strongly institutionalised, the break with these structures cleared the way for a decentralised and simple but appropriate technical and organisation solution.

Thus, one strategy for the public sector can be to move some of its own ICT services to new platforms where the users already are part of the installed base and not to try to “force” the users to use only the existing information infrastructure which is controlled by the government. 

## 6. Conclusion

This paper has presented a three-year development project, in which changes in professional health service provision along with system development work and changes in the organization have been closely woven together. The decision to implement a new health treatment model entailed organizational changes and a move from the dominating technical and organizational infrastructure to a new mobile platform including the development of an application using (smart) mobile phones. We claim that this departure from the existing centralised institutional framework to a more independent, decentralized treatment model made this shift easier. At the same time, the user-oriented innovation process seems to have helped the implementation of the treatment model and also to have stimulated the growth of a new installed base, as it was directly linked to the new organizational model supporting decentralized healthcare delivery through an alternative technical platform within an existing and simpler infrastructure. In our analysis of the user innovation in an institutionalized environment, we have illustrated that theory from information infrastructure, system development, and user-driven innovations can be combined to understand how and why the project succeeded. The experiences from this case support the view that user-centred, bottom-up innovations can replace or at least supplement top-down controlled development work in the health sector. From a political point of view, closer cooperation between health care workers and patients is emphasized as important in order to ensure better health care services. Health care workers seek to understand the user's needs better and in more detail. 

Our case shows that making an incremental, user-driven innovation through small clusters of users and building organizational networks are one way by which to surmount the barriers associated with existing technical and institutional structures, the dominating installed base. One conclusion is that new organizational structures supported by new technical component should seek to benefit from an existing but adequate installed base. This may then trigger gradual changes in parts of the existing organizational structure and make possible to build links between the old and new information infrastructures. More research is, however, needed to understand how specific characteristics of the local technical, organisational, and cultural context influence such technology innovation and diffusion processes.

## Figures and Tables

**Table 1:** Research framework.

Analytic level	Focus in the analysis	Critical factors/processes
Organizational level: health service provision and so forth.	Identifying the organizational changes processes and reform(s) in the psychiatric health service provisions that have taken place, including changes in work patterns, relations between professionals and the user, and furthermore, institutional and professional interests and conflicts.	Context: institutional variables, changes in organizations, important actors and power structures, and professional interests and conflicts
The application development and user level	Identifying and understanding all phases in the system development process and how they involved various user groups which had different background, experience, and interests in the this work	System development approaches and phases. Different actors and roles in system development
Information infrastructure level (II)	Understanding the specific characteristics of the different II and their installed base; how they influenced the change processes at different levels (technical, organisational). More specifically: what made it possible to move from an old to a new II?	Characteristics of the two II/IB. Technical platform, standards, basic functions services, dynamics, type of applications typical users

**Table 2:** Data collections methods used.

Methods	Type of activities				Total
	2006	2007	2008	
Observation (during participation in meetings)	3 project teams 7 steering groups	7 project teams 3 steering groups 5 contractors	4 project teams 3 steering groups 5 techno groups	other meetings	63
Observation (user courses)			3 observations		3
Interviews	12 from ambulant teams	4 user representatives			16
Questionnaire			2 questionnaire		2
Literature	Project documents	Meeting notes, e-mail, and reports	user-training notes, workshop documentation	Other documents	<100

**Table 3:** Stakeholders, phases, and roles in the system development work.

Stakeholders	The different phases and roles in the system developing work								
	Analyze, start, meetings	Choice of method	Design development	Choice of technology	Testing mobile solution	Implement- ation	Courses training	Course user partici- pating	Evaluating
Children and youth									X
Parents	X		X	X	X		X	X	X
Ambulant team	X	X	X	X	X	X	X	X	X
CYP leaders	X	X		X	X	X	X		X
Clinic superior	X	X		X		X			X
Health enterprise leaders				X		X			
Project group	X	X	X	X	X	X	X	X	X
Steering group	X	X		X		X		X	X
Reference group							X		X
Techno group			X						
User organizations	X	X	X	X	X	X	X	X	X
Regional ICT unit	X								
Local ICT unit	X								
Supplier			X		X		X		
Superior for purchase	X								
Service workers in the community								X	X

**Table 4:** Framework for old and new regime/technical and organisational model.

Model				
Level	Old regime/technical and organizational model		New regime: technical and organizational model	
	Old organisational structure	Old system development model	New organisational structure	New system development model
Changes in organization and health service provision	Centralized treatment model	Traditional SU methodology: top-down, expert driven Organised at NCT (Norwegian Centre for Telemedicine)	PMT-O: decentralized	Local, user-oriented, incremental and experimental system development Local project group (techno group), local health personnel + users
Development of the applications	Application based on PC and videoconferences		Application based on mobile phone	
Infrastructure	II/IB: based on Broadband Norw. Health Network		Mobile telephone network. Establishment of local techno group	
